# Lactoferrin Decreases Enterotoxigenic *Escherichia coli*-Induced Fluid Secretion and Bacterial Adhesion in the Porcine Small Intestine

**DOI:** 10.3390/pharmaceutics14091778

**Published:** 2022-08-25

**Authors:** Matthias Dierick, Ruben Ongena, Daisy Vanrompay, Bert Devriendt, Eric Cox

**Affiliations:** 1 Laboratory of Immunology, Faculty of Veterinary Medicine, Ghent University, 9820 Merelbeke, Belgium; 2 Laboratory for Immunology and Animal Biotechnology, Faculty of Bioscience Engineering, Ghent University, 9000 Ghent, Belgium

**Keywords:** *Escherichia coli*, lactoferrin, postweaning diarrhea, pigs

## Abstract

Enterotoxigenic *Escherichia coli* (ETEC) infections are one of the most prevalent causes of post-weaning diarrhea in piglets, resulting in morbidity, mortality and elevated use of antibiotics. The emergence and further spread of antimicrobial resistance together with the growing demand for high quality animal protein requires the identification of novel alternatives for antimicrobials. A promising alternative is lactoferrin, as we previously showed that it can both inhibit the growth and degrade bacterial virulence factors of porcine ETEC strains in vitro. Aiming to confirm these findings in vivo, we performed a small intestinal segment perfusion experiment in piglets. Here, we showed that lactoferrin could not only decrease ETEC-induced fluid secretion, but also their ability to colonize the small intestinal epithelium. Furthermore, while ETEC infection induced pro-inflammatory cytokine mRNA expression in this experiment, lactoferrin was not able to counteract these responses. In addition, a bacterial motility assay showed that lactoferrin can reduce the motility of ETEC. Our findings further support the use of lactoferrin as an alternative for antimicrobials and also show its potential for the prevention of ETEC infections in pigs.

## 1. Introduction

One of the most common agents associated with post-weaning diarrhea (PWD) in piglets is enterotoxigenic *Escherichia coli* (ETEC) [1,2]. ETEC infections mostly affect piglets in the first two weeks after weaning, resulting in a watery diarrhea leading to dehydration, growth retardation and sometimes mortality. A crucial factor in the onset of this disease is the production of virulence factors by ETEC, such as the heat stable enterotoxins STa and STb, the heat labile enterotoxin LT and fimbriae [3]. In pigs, ETEC-induced PWD is mostly associated with F4- and F18-fimbriated *Escherichia coli (E. coli)*. These F4 and F18 fimbriae are filamentous polymeric proteins that interact with specific glycosylated receptors, present on the surface of small intestinal epithelial cells [4,5,6]. The most common way to control PWD is the extensive use of antibiotics and zinc oxide, which have most likely contributed to the increased presence of multi-antibiotic resistant strains [7,8,9]. The rapid appearance of multi-drug resistant bacterial strains is very concerning, especially the appearance of antibiotic resistance against the so-called last-resort antibiotics, which are reserved for the treatment of multi-resistant bacterial infections in humans [10]. In addition, the European Union banned the use of zinc oxide in 2022, showing the urgency to search for good alternatives for use in food-producing animals, such as pigs [11,12]. Several governmental action plans on combatting antimicrobial resistance (AMR) are boosting the research and development of new alternatives, aiming to decrease antibiotic use [13,14]. One of these alternatives are naturally derived proteins, such as lactoferrin (LF), which are not only capable of directly affecting the microorganisms, but also enhancing host immune responses against them [15,16].

LF is a glycoprotein belonging to the family of non-haem iron-binding proteins and is present in several mammalian secretions, including those of pigs and humans [15,17]. It is a moonlighting protein displaying a wide variety of activities, such as iron homeostasis, cellular growth and differentiation, antimicrobial activities, immunomodulatory activities and antiviral activities [16,18,19,20,21]. In addition, LF has recently emerged as a potential antiviral treatment in COVID-19 management [22,23]. The antibacterial activity of LF can be divided into (1) a bacteriostatic activity via its chelation of Fe^3+^ ions, hereby depriving bacteria of this essential nutrient and (2) a bactericidal activity through its interaction with lipopolysaccharides (LPS), causing a disruption of the bacterial cell wall [24]. Previously, we have shown that incubation of LF with enterohemorrhagic *E. coli* (EHEC) O157:H7 and several porcine ETEC strains inhibited the growth of these pathogens [25,26]. Furthermore, LF is also capable of inhibiting the attachment of pathogens, such as *Chlamydia psittaci* (*C. psittaci*), EHEC and ETEC, to chicken macrophages and intestinal epithelial cells, respectively [25,26,27]. Besides these properties, LF also possesses a proteolytic activity towards a diverse array of bacterial virulence factors [25,28,29]. For example, LF is capable of cleaving EHEC-associated virulence factors, including the receptor-binding unit of Shiga toxin-2 as well as EspA and EspB, components of the type III secretion system, which are important for the adherence of EHEC to intestinal epithelial cells [25,29,30]. Furthermore, LF can cleave the Hap adhesin of *Haemophilus influenzae* (*H. influenzae*) at an arginine-rich site, more specifically VRSRRAAR [28]. Previously, we have identified that LF can degrade ETEC-associated virulence factors, such as F4 and F18 fimbriae [26]. Inhibiting the proteolytic activity of LF using a serine protease inhibitor indicated that the fimbrial degradation by LF plays an important role in reducing the adhesion of ETEC to intestinal epithelial cells under in vitro conditions. Additionally, LF was also able to degrade flagellin; however, the effect on motility of ETEC remains unclear [26].

The antibacterial and proteolytic activity of LF has been well-defined in vitro, however its effect upon infections in weaned pigs requires further elucidation. Previous in vivo studies in neonatal pigs focused on the effect of LF on the mucosal and systemic immune responses [31,32]. Dietary bovine LF (bLF) was shown to increase serum Immunoglobulin G (IgG) levels and lactoferricin-lactoferrampin-encoding *Lactobacillus reuteri* and *Lactococcus lactis* were also able to increase intestinal secretory Immunoglobulin A (IgA) levels [31,32,33]. Furthermore, LPS-induced tumor necrosis factor α (TNF-α) and interleukin (IL) 1β mRNA expression by IPEC-J2 cells is decreased in the presence of the LF-producing lactobacilli [32]. Here, we assessed the effect of both bLF and recombinant porcine LF (pLF) on their ability to ameliorate ETEC-induced symptoms in an in vivo setting. BLF was used as it easily accessible and often used in LF studies. pLF was also included as previous research indicated that LF from one species is more effective against pathogens of that species. For instance, bLF was more effective in inhibiting growth of EHEC O157:H7 and their attachment to intestinal epithelial cells, compared to human LF [25]. Furthermore, ovotransferrin, compared to human LF and bLF, was shown to be more effective in inhibiting attachment and entry of *C. psittaci* in chicken macrophages [27].

In this study, we aimed to examine whether our previous in vitro results on the adhesion of ETEC bacteria to gut epithelial cells and the degradation of ETEC virulence factors, could ameliorate ETEC-induced diarrhea in vivo. Using small intestinal segment perfusion experiments (SISPs), we evaluated the effect of LF on the ETEC-induced fluid absorption, the expression of immune-related genes as well as on the adhesion of ETEC to the small intestinal epithelium.

## 2. Materials and Methods

### 2.1. Bovine and Porcine Lactoferrin

bLF, derived from bovine milk, with a 92% purity and 16% iron saturation (as determined by the manufacturer) was purchased from Ingredia Nutritional (Arras, France). pLF was produced in-house by Chinese Hamster Ovary (CHO) cells, using a workflow developed by our laboratory, and purified as previously described [26,34]. Briefly, recombinant pLF in CHO suspension culture medium was purified using a HisTrap^TM^ HP column (Cytiva, Marlborough, MA, USA) and a stepwise gradient of an imidazole-containing elution buffer. Finally, the eluted proteins were dialyzed against phosphate-buffered saline (PBS) overnight at 4 °C and the purity and iron saturation were determined using silver staining and measuring the A_280_/A_466_ ratios, respectively, as previously described [26,35].

### 2.2. Bacterial Strains

Different porcine ETEC strains were used in this study. In the SISP experiment the F18-fimbriated 2134P ETEC strain (O157:H19, Sta^+^ STb^+^, flagellin^+^) was used (Tiels et al., 2005, Vet. Mic.), while in the bacterial motility assay the F4-fimbriated ETEC strains GIS26 (O149:K91, LT^+^ Sta^+^ STb^+^, flagellin^+^) and IMM01 (O149:K91, LT^+^ STb^+^, flagellin^−^) were used (Verdonck et al., 2004, Vet. Mic.) [36,37].

### 2.3. Animals

Twelve 4-week-old newly weaned, conventionally reared piglets (Landrace x Piétrain) were selected to be seronegative for F18 fimbriae-specific antibodies. Furthermore, the selected animals were F18 receptor positive, as assessed by polymerase chain reaction—restriction length polymorphism (PCR-RFLP) on the α(1,2)fucosyltransferase 1 (FUT1) gene [38]. Animals were transported to the faculty and were randomly assigned to two groups: a bLF group (*n* = 6) and a pLF group (*n* = 6). Both groups were housed in the same isolation unit. This experiment was reviewed and approved by the Ethical Committee of the Faculties of Veterinary Medicine and Bioscience Engineering at Ghent University, in accordance with the Belgian law on animal experimentation (EC2019/085—approved 22 June 2019).

### 2.4. Small Intestinal Segment Perfusion Assay

The small intestinal segment perfusion model (SISP) [39] was performed to assess the effects of bLF and pLF on (1) intestinal fluid secretion, (2) ETEC adhesion and (3) the transcript levels of innate immune genes in the small intestine. Piglets were fasted overnight. The next morning, premedication was administered by intramuscular injection of 0.75 mg/kg midazolam (Mylan, Hoeilaart, Belgium), 15 mg/kg ketamine (Ecuphar, Oostkamp, Belgium) and 0.15 mg/kg morphine (Sterop, Brussels, Belgium). After 20 min, anesthesia was induced by intravenous (IV) injection of 2–4 mg/kg propofol (Zoetis, Zaventem, Belgium). Subsequently, piglets were intubated in the endotrachea and maintained under long-term anesthesia with a mixture of 1.5% isoflurane (Zeotis, Zaventem, Belgium) and 40% oxygen (Air Liquide, Luik, Belgium). Hematocrit values (Hct) were assessed regularly and when exceeding 35, 10–15 mL/kg/h ringer lactate (Baxter, IL, USA) was infused via the ear vein to prevent dehydration. Temperature, heart rate, oxygen saturation, expiratory CO_2_ and non-invasive blood pressure were monitored continuously throughout the experiment.

The surgical and experimental procedures have been previously described in detail [39]. Briefly, the abdomen was opened at the linea alba and six small intestinal segments were constructed in the mid-jejunum starting at a distance of 200 cm distal to the ligament of Treitz. Segments were 10 cm in length with a distance of 5 cm between each segment. These segments retained their vascularization and were cannulated with a rubber tube at the proximal and distal ends to inject and collect fluid, respectively.

Intestinal segments were perfused with (1) 5 mL of perfusion fluid (0.9% NaCl + 0.1% glucose), (2) 5 mL of perfusion fluid containing 8 × 10^8^ colony forming units (CFU)/mL STa^+^ STb^+^ F18ac bacteria (strain 2134P) [36] or (3) 5 mL of perfusion fluid containing 8 × 10^8^ CFU/mL Sta^+^ STb^+^ F18ac bacteria pre-incubated for 1 h at 37 °C with 4 different concentrations of bLF or pLF (50, 25, 10 and 5 mg). The experiment ran for 5 h and every 15 min, 2 mL perfusion fluid was injected, whereafter piglets were euthanized with an overdose of sodium pentobarbital (Kela Laboratoria, Hoogstraten, Belgium). Upon euthanasia, outflow was collected and the weight of the residual fluids in the segments was measured. Additionally, the length and width of each segment was measured to calculate the intestinal surface area (cm^2^). The net fluid absorption was calculated as: (input-output)/surface area (g/cm^2^). Furthermore, the segments were excised and washed 3 times with ice-cold PBS after which tissue samples from each segment were collected and frozen for RNA isolation and immunohistochemistry.

### 2.5. RT-qPCR

Total RNA was extracted from approximately 100 mg frozen intestinal tissue by homogenizing the tissue in 1 mL TRIzol^®^ Reagent (Invitrogen, Merelbeke, Belgium). RNA concentration, quality and purity was measured using a Nanodrop^®^ ND-1000 (DeNovix, Wilmington, DE, USA). Only samples with a 260/280 ratio between 1.8–2.1 and 260/230 ratio between 1.5–2.0 were used for further analysis. RNA integrity was checked using agarose gel electrophoresis on a 2% Tris-Acetate-EDTA (TAE) gel before proceeding to conversion of RNA to cDNA. Total RNA of each sample was subjected to DNase treatment (RQ1 RNase-Free DNase; M6101; Promega, Madison, WI, USA) according to the manufacturer’s protocol. Subsequently, RNA (1 µg) was reverse transcribed into single stranded cDNA using the SuperScript III Reverse Transcriptase kit (Invitrogen) in presence of a recombinant ribonuclease inhibitor (RNase OUT; Invitrogen) according to the manufacturer’s instructions to obtain cDNA in a single-step reaction. This reaction consists of 5 min at 25 °C (priming), 50 min at 50 °C (reverse transcription) and 15 min at 70 °C (RT inhibition). Resulting cDNA served as a template for the quantitative polymerase chain reaction (qPCR) assay. Primer oligonucleotides were synthesized by Integrated DNA Technologies (IDT, Coralville, IA, USA). The sequences and concentrations of the forward and reverse primers used are mentioned in Table 1. For some genes, new primers were designed using the primer design tool PrimerBLAST (NIH, Bethesda, MD, USA). Next, a SensiFAST™ Hi-ROX SYBR^®^ green PCR Master Mix (Bioline, Meridian Bioscience, Cincinatti, OH, USA) was used for quantitative PCRs (qPCRs) using 1 µL (50 ng) cDNA and the forward and reverse primer from each target gene, following the manufacturer’s protocol. After preparing the master mix (final volume = 20 µL) in MicroAmp Fast optical 96-well-reaction plates (Applied Biosystems, Thermo Fisher Scientific, Waltham, MA, USA), qPCRs were performed using a StepOne Plus real-time PCR system (Applied Biosystems, Thermo Fisher Scientific, Waltham, MA, USA). Thermal cycling conditions were 1 cycle of 10 min at 95 °C followed by 40 cycles of 15 s at 95 °C, 30 s at the annealing temperature (60 °C), and 30 s at 72 °C. Melting curve analysis confirmed primer specificities and all reactions were run in duplicate. The relative expression level of the target genes was analyzed by the double delta threshold cycle method and normalized to the expression level of the reference genes Glyceraldehyde-3-phosphate dehydrogenase (GAPDH) and Ribosomal Protein L19 (RPL-19) and to the control condition (perfusion fluid).

### 2.6. Immunohistochemistry

Upon washing the intestinal segments, these were embedded in 2% (*w*/*v*) methocel (Fluka, Bornem, Belgium), snap-frozen in liquid nitrogen, and stored at −80 °C. Frozen tissue was then cut into cryosections (8 µm) using a cryotome (Leica CM3050 S, Leica Biosystems, USA, IL) and mounted on 3-aminopropyl-triethoxysilane (APES, Sigma-Aldrich, St. Louis, MO, USA) coated glass slides. Next, sections were dried for 2 h at 60 °C, fixed in acetone at −20 °C for 10 min and stored at −20 °C. After air-drying the sections for 1 h at room temperature, sections were incubated with 50 mM ammonium chloride (pH 8.0) for 30 min followed by several washes with PBS. Sections were then blocked with 10% goat serum in PBS for 30 min in a humid cell chamber at 37 °C. Upon blocking, the sections were consecutively incubated with: F18 fimbriae-specific polyclonal rabbit serum (1:10 dilution; in-house) and fluorescein isothiocyanate (FITC)-conjugated secondary anti-rabbit antibody (1:100 dilution; Sigma-Aldrich, St. Louis, MO, USA) for 1 h at 37 °C. Sections were washed with PBS in between steps, and a Hoechst counterstain (10 µg/mL) was applied for 2 min to visualize cell nuclei. The sections were mounted in glycerol with 0.223 M 1,4 diazobicyclo (2,2,2)-octane (DABCO; Sigma-Aldrich, St. Louis, MO, USA) to prevent photobleaching. The mounted sections were visualized using a fluorescence microscope (Leica, Wetzlar, Germany). Images were processed as follows in Fiji: first, the intestinal tissue was delineated based on the Hoechst signal and then the mean fluorescence intensity (MFI) in the FITC channel was quantified in each selection. Then, the MFI of each section was corrected for the total surface area of the tissue (MFI/mm^2^). Lastly, the MFI in the FITC channel was compared across control-, ETEC- and ETEC + 50 mg bLF or pLF-perfused segments.

### 2.7. Bacterial Motility Assay

To assess if lactoferrin can reduce bacterial motility, a flagellated (LT^+^ STa^+^ STb^+^ F4ac bacteria, GIS26) and a non-flagellated (LT^+^ STb^+^ F4ac bacteria, IMM01) porcine ETEC strain [40] were used. A colony of these strains was cultured in 5 mL Tryptone Soy Broth (TSB) medium for 18 h at 37 °C with shaking (180 rpm). The overnight cultures were then subcultured 1/50 in TSB at 37 °C with shaking (180 rpm) containing different concentrations of bLF (0, 0.5 and 1 mg/mL) until an Optical Density (OD)_600_ = 0.5 was reached. Next, 1 µL of these cultures was stabbed into the middle of a TSB 0.3% agar Petri dish. After 8 h incubation at 37 °C, images were taken and the surface area of the motility ring was calculated using Fiji.

### 2.8. Data Analysis

Statistical analysis of data was performed using GraphPad Prism 9. Net absorption of fluid by gut segments in the SISP model was analyzed using a Friedman test with a correction for multiple comparisons by controlling the false discovery rate using the two-stage linear step-up procedure of Benjamini, Krieger, and Yekutieli. RT-qPCR results were normalized to the control segment and subsequently analyzed using a Wilcoxon matched-pairs signed rank test to compare ETEC-perfused segments to control-perfused segments and a Kolmogorov–Smirnov test to compare ETEC + 50 mg bLF/pLF- perfused with ETEC-perfused segments. Outliers were identified using ROUT’s method (Q = 1%) and subsequently removed from RT-qPCR analysis. Mean fluorescence intensity was quantified in the immunohistochemistry experiment and was analyzed using a Wilcoxon matched-pairs signed rank test. The data of the bacterial motility assay were first checked for normality using a Shapiro–Wilk normality test and subsequently analyzed using one-way ANOVA with Tukey correction for multiple comparisons.

## 3. Results

### 3.1. LF Attenuates the ETEC-Induced Reduction in Intestinal Fluid Absorption

Our previous work revealed the ability of bLF and pLF to disarm the virulence factors of porcine ETEC strains, by degrading F4 and F18 fimbriae and decreasing the ability of these strains to adhere to porcine enterocytes [26]. Building further on these findings, we questioned whether LF could also reduce ETEC-induced diarrhea in piglets.

**Figure 1:**
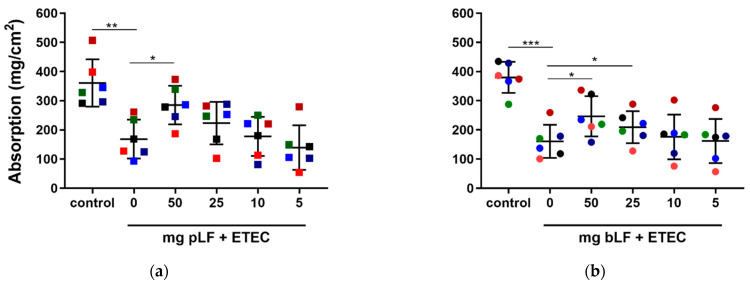
LF attenuates ETEC-induced reduction in fluid absorption in the SISP model. Jejunal segments were incubated with either control (perfusion fluid), F18^+^ ETEC and (**a**) F18^+^ ETEC with four different concentrations of pLF or (**b**) F18^+^ ETEC with four different concentrations of bLF. Individual data points represent different animals, whereas the horizontal line represents the mean and the error bars the standard deviation (*n* = 6). *, q  <  0.05; **, q < 0.01; ***, q < 0.001. bLF, bovine lactoferrin; pLF, recombinant porcine lactoferrin, the pigs are individually colored and the squares or circles represent treatment with bLF or pLF, respectively.

To assess this, we performed SISP experiments in which the absorption of the perfusion fluid in each segment perfused with either perfusion fluid (control), ETEC and different concentrations of recombinant pLF or bLF (50, 25, 10 and 5 mg) together with ETEC was determined. As shown in Figure 1, segments perfused with F18^+^ ETEC displayed a significantly decreased net absorption as compared to the control segments in the pLF (q = 0.0011) and bLF (q = 0.0002) groups. In contrast, the highest amount of pLF tested (50 mg) attenuated the ETEC-induced reduction in fluid absorption (q = 0.0433) (Figure 1a). Likewise, perfusion of the segments with ETEC in the presence of 50 and 25 mg bLF attenuated the ETEC-induced reduction in fluid absorption (q = 0.0213) and (q = 0.0471), respectively (Figure 1b).

### 3.2. ETEC Infection Induces Expression of Innate Immune Genes in the Gut

**Figure 2:**
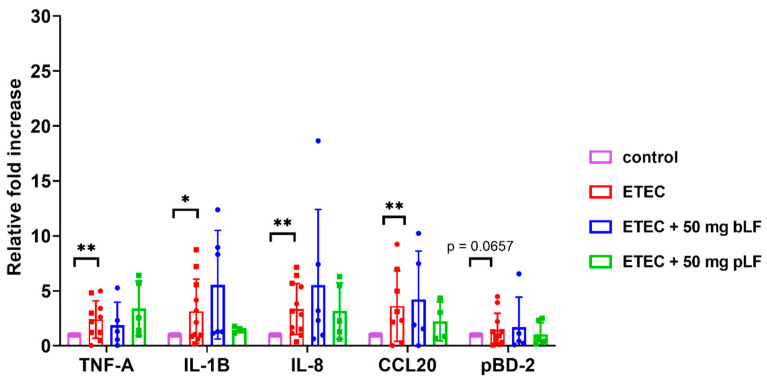
LF does not affect ETEC-induced upregulation of innate immune genes in the gut tissues. Transcript levels of genes in jejunal segments perfused with F18^+^ ETEC and segments perfused with ETEC + 50 mg pLF or 50 mg bLF were compared to control segments. Individual data points represent different animals, whereas the horizontal line represents the mean and the error bars the standard deviation (*n* ≥ 5). *, *p* < 0.05; **, *p* < 0.01. bLF, bovine lactoferrin; pLF, recombinant porcine lactoferrin.

ETEC is known to trigger the expression of genes related to the intestinal innate immune system in piglets [39]. To investigate whether bLF and pLF could affect ETEC-induced changes in the transcriptional landscape, mRNA expression of the pro-inflammatory cytokines TNF-α and IL-1β, the chemokines IL-8 (attracts neutrophils) and chemokine (C-C motif) ligand 20 (CCL20) (attracts dendritic cells) and the host defense protein porcine β-defensin 2 (pBD-2) was assessed in the small intestinal segments. As the strongest attenuation of the ETEC-induced reduction in fluid absorption was observed with the highest dose of bLF and pLF (50 mg), only segments from these conditions were considered relevant to assess the effects of LF on the transcriptional landscape. The results show a significant upregulated mRNA expression of TNF-α, IL-1β, IL-8 and CCL20 after infection with ETEC as compared to the control condition across both groups (Figure 2). Interestingly, pLF and bLF did not affect the ETEC-induced upregulation of transcript levels of the examined genes (Figure 2).

### 3.3. bLF and pLF Decrease Adhesion of F18^+^ ETEC to the Small Intestinal Epithelium

**Figure 3:**
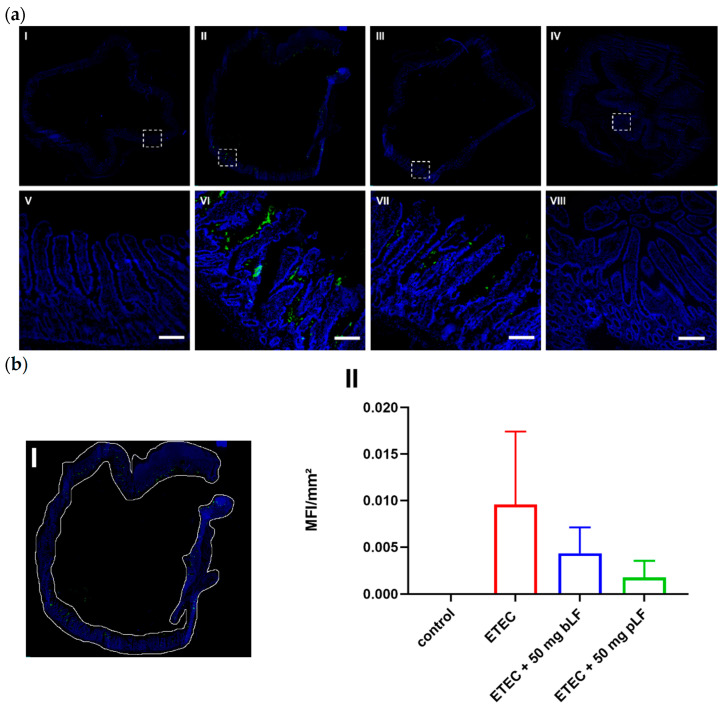
bLF and pLF decrease ETEC adherence to the small intestinal epithelium. Cryosections of jejunal segments perfused with perfusion fluid (control), ETEC and ETEC + 50 mg bLF or pLF were stained with F18 fimbriae-specific antibodies to detect the presence of F18^+^ ETEC (in green). Nuclei were counterstained with Hoechst (blue). (**a**) Representative image of segments perfused with perfusion fluid (control) (**I**), ETEC (**II**), ETEC + 50 mg bLF (**III**) and ETEC + 50 mg pLF (**IV**). The white dashed squares highlight areas, which are shown in the bottom images (**V**–**VIII**). (**b**) (**I**) Shows how the tissue was selected to determine the mean fluorescence intensity (MFI) in the FITC channel in each segment. (**II**) Data are presented as the mean ± the standard deviation (*n* = 2–5). bLF, bovine lactoferrin; pLF, porcine lactoferrin; Scale bar = 200 µm.

Previously, we showed that LF decreased the adhesion of porcine ETEC to intestinal epithelial cells [26]. In order to assess whether bLF and pLF are able to reduce ETEC adhesion in vivo, gut tissue from the segments were stained to detect the presence of F18 fimbriae. As shown in Figure 3a, ETEC bacteria were observed in close proximity to the small intestinal epithelium in segments perfused with ETEC. As expected, control segments were completely negative. Interestingly, both bLF and pLF seemed to be able to reduce ETEC adhesion to the epithelium, as a less intense signal was observed. To quantitate these findings, the entire tissue was selected (Figure 3(bI)) and the mean fluorescence intensity (MFI) was determined. Control segments completely lacked a fluorescent signal (Figure 3(bII)). In contrast, gut tissues of the ETEC-perfused segments displayed a strong fluorescent signal, indicating adherence of F18^+^ ETEC, while segments perfused with ETEC and 50 mg bLF or pLF showed nearly a 50% decrease in fluorescent signal (Figure 3b). This implies that both bLF and pLF reduced ETEC adhesion to the porcine small intestinal epithelium.

### 3.4. bLF Reduces Bacterial Motility of ETEC

**Figure 4:**
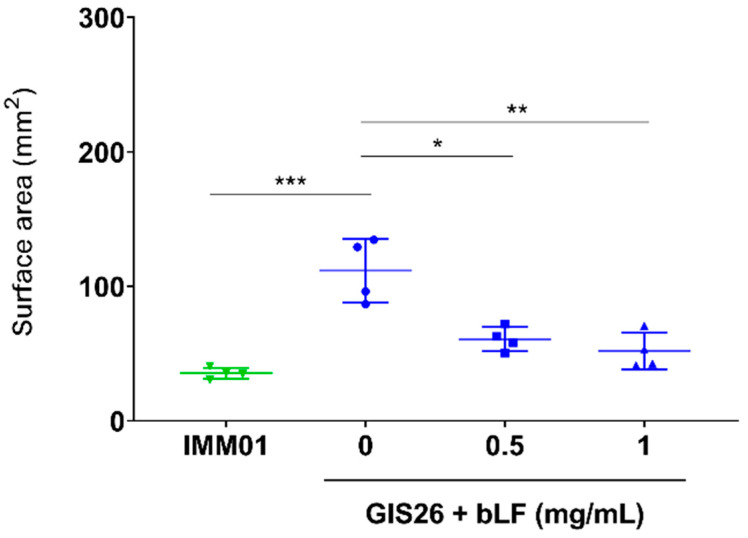
bLF reduces the motility of ETEC. The flagellated ETEC strain GIS26 was incubated with different concentrations of bLF, whereas the non-flagellated ETEC strain IMM01 was used as a negative control. The surface area (mm^2^) of the motility ring was measured using Fiji. The horizontal line shows the mean and the error bars the standard deviation (*n* = 4). *, *p*  <  0.05; **, *p* < 0.01; ***, *p* < 0.001. bLF, bovine lactoferrin.

In our previous study, we showed that LF can degrade flagellin, a key component of the molecular machinery involved in bacterial motility [26]. Since the reduced adhesion of ETEC observed above might result from an impaired motility of ETEC, the ability of LF to affect ETEC motility was investigated. As expected, the motility of the flagellated ETEC strain (GIS26) differed significantly from the non-flagellated ETEC strain (IMM01) (Figure 4). Furthermore, upon incubation of the GIS26 strain with 0.5 and 1 mg/mL bLF, a significant decrease in motility, almost to the level of the non-flagellated strain, could be observed compared to the control condition (Figure 4). This shows that bLF can affect bacterial motility, most likely via its ability to degrade flagellin.

## 4. Discussion

Post-weaning diarrhea is an economically important disease in the worldwide pig industry, mainly affecting piglets in the first two weeks after weaning. The most common agents associated with PWD in piglets are F4- and F18- fimbriated ETEC strains producing enterotoxins [1]. The latter trigger excessive fluid secretion in the intestine, causing diarrhea, which in turn leads to dehydration, growth retardation and even mortality. Antibiotics are often used to control ETEC infections, however, the emergence and spread of multi-resistant strains warrants the development of novel alternatives [41,42]. LF is such an alternative as its antibacterial activity has already been described [43].

Here, we used a SISP model, optimized in our lab, to assess the effect of LF on the ETEC-induced fluid secretion [39,44]. ETEC infection resulted in a reduced fluid absorption, while the addition of bLF and pLF attenuated this disruption of the intestinal water balance. Interestingly, bLF seemed to be more effective as it was capable of attenuating this disruption at a lower concentration (25 mg) than pLF (50 mg). The reason for this remains unclear as previous research did not show differences in the ability of bLF and pLF to degrade virulence factors and to decrease epithelial attachment of ETEC [26]. The recombinant production of pLF in CHO cells might introduce subtle changes to its structure, potentially caused by a different glycosylation pattern, as compared to native pLF, that might affect its in vivo behavior [45,46,47,48]. Further research will be needed to clarify this.

Lactoferrins are known for their immunomodulatory activities. The latter is, in part, dependent on the direct interaction of LF with pathogen-associated molecular patterns (PAMPs), such as LPS [16,21,49]. Indeed, LF can block the binding of LPS to LPS binding-serum protein and soluble Cluster of Differentation (CD14), hereby attenuating the production of pro-inflammatory cytokines through Toll-Like Receptor 4 (TLR4) signaling [16,21]. Thus, in addition to evaluating the effect of LF on the ETEC-induced fluid secretion, we investigated the impact of LF on ETEC-induced changes in the expression of immune-related genes. Several cytokines and chemokines have been shown to be upregulated in gut epithelial cells upon ETEC infection, both in vitro and in vivo [32,39,40,50]. Here, an upregulated mRNA expression of the pro-inflammatory cytokines TNF-α and IL-1β as well as the chemokines IL-8 and CCL20 was observed upon ETEC infection. However, the administration of LF did not affect these ETEC upregulated genes. This indicates that ETEC-derived virulence factors other than LPS, such as flagellin or enterotoxins, might be important for the upregulation of these cytokines and chemokines. This corresponds with previous in vitro observations where IL-8 was upregulated upon incubation of porcine small intestinal epithelial cells, either as a cell line (IPEC-J2) or gut organoids, with ETEC or its enterotoxins [40,51]. This together with our findings presented here indicate that ETEC-derived enterotoxins or flagellin might be important in driving cytokine and chemokine secretion by the gut tissues. For instance, flagellated bacteria, such as *E. coli*, can promote the production of IL-17C and IL-8 by epithelial cells, through TLR5 signaling [52,53,54]. However, flagellin can be degraded by LF but this might not affect its ability to activate TLR5-mediated signaling maintaining its ability to trigger inflammatory responses in gut epithelial cells and immune cells. Further research is needed to clarify whether the LF-mediated degradation of flagellin affects TLR5 signaling pathways. Although LF does not impact ETEC-induced upregulation of immune-related genes, this might be beneficial for the host as it allows to mount innate and adaptive immune responses required to clear the infection.

On the other hand, we need to take into account the limited time frame of the SISP experiments, which could be insufficient to evaluate the impact of LF on the ETEC-induced changes in the transcriptional landscape. This is relevant when considering the interaction of LF with its receptor intelectin, which is expressed in the small intestine. Binding of LF to intelectin triggers its uptake and subsequent translocation to the nucleus [16,55]. Interestingly, infection of bovine rectal epithelial cells with EHEC resulted in an increased translocation of bLF to the nucleus [56]. Furthermore, an alternative splice variant of human LF, Δlactoferrin, also translocates to the nucleus, where it can interact with a ∆lactoferrin response element (∆LFRE), characterized by the consensus sequence GGCACTT(A/G)(C/G) [55,57,58]. The ∆LFRE can be found upstream of several genes, such as *Skp1*, *Bax*, *Fas* and *DcpS*, and the direct interaction between ∆LF and ∆LFRE was confirmed for these targets, using Chromatin Immunoprecipitation (ChIP) experiments [58,59]. This implies that LF could alter the expression of immune-related genes. Currently, it is unknown whether nuclear translocation of bLF and pLF occurs in porcine small intestinal cells. However, this experiment was only conducted in a 5 h window and this could be insufficient for LF to translocate to the nucleus and to alter gene expression. Further experiments will be required to determine whether LF can translocate to the nucleus of porcine intestinal epithelial cells and which gene transcription is altered by LF and whether these changes allow LF to modulate the innate and adaptive immune responses upon ETEC infection in piglets.

As LF attenuated the ETEC-induced reduction in fluid absorption and since our previous results demonstrated that LF degrades both the structural subunit FedA and the tipadhesin FedF of F18 fimbriae [26], we hypothesized that this might be due to a decreased adhesion of F18-fimbriated ETEC to the intestinal epithelium. Indeed, we showed that both bLF and pLF reduced the adhesion of ETEC to the intestinal epithelium. In addition to ETEC, LF also decreases the adhesion of other pathogens, such as EHEC, Streptococcus dysgalactiae and C. psittaci, to their target cells. This could be explained by the degradation of virulence factors involved in adhesion of these organisms. Some of the virulence factors that are degraded by LF are: EspA and EspB of EHEC bacteria and Hap adhesin of H. influenzae. Although these virulence factors are all degraded by the proteolytic activity of LF, still no common consensus sequence has been identified which is targeted by LF [25,26,27,28,60]. Nevertheless, LF-mediated degradation of virulence factors playing a role in pathogen adhesion seems to emerge as a common theme in the antimicrobial activities of lactoferrins. It might be of interest to investigate whether lactoferrins degrade colonisation factors of other pathogens as well. In addition to the degradation of F4 and F18 fimbriae, bLF and pLF are also able to degrade flagellin, the main component of flagellae which control bacterial motility [26]. Here, we showed that bLF can decrease ETEC motility. This implies that degradation of flagellin by bLF could also lead to a decreased motility of *E. coli* in the gut [61]. Therefore, this could impede colonisation of the small intestine by ETEC bacteria. Although the role of flagellin in ETEC colonization of the small intestine is still unclear, flagella-driven motility could play an important role in the onset of PWD, as it is involved in bacterial adhesion to intestinal epithelial cells [61,62].

Altogether, the experiments conducted in this manuscript provided us with interesting new insights in the interaction of LF with porcine ETEC. While LF did not decrease the mRNA expression of pro-inflammatory cytokines triggered by ETEC infection, it does seem to attenuate the ETEC-induced reduction in fluid absorption, possibly by decreasing ETEC adhesion to the epithelium. This, in turn, could be explained by either LF-mediated degradation of fimbriae or a decreased motility of ETEC by the degradation of flagellin. To identify the most important underlying mechanism further research is warranted. In addition, our research findings further support the possible use of bLF as a feed additive to further decrease antibiotic usage in pigs to decrease PWD caused by multi-resistant ETEC strains.

## 5. Conclusions

In conclusion, LF ameliorates the ETEC-induced reduction in fluid absorption, most likely by decreasing ETEC adherence to the epithelium and its motility in the gut lumen. This indicates that LF as a feed additive might result in less severe symptoms of PWD in piglets. These findings further support the potential of LF as an alternative strategy to protect against infection with gut-dwelling bacterial pathogens.

## Figures and Tables

**Table 1:** Sequences and concentrations of the used primers.

Gene *	Sequence (5′ → 3′)	Size (bp)	Primer Conc. (µM)	Tm (°C)
TNF-α	F:ACTGCACTTCGAGGTTATCGG	118	250	60
	R:GGCGACGGGCTTATCTGA		250	
IL-1β	F:GAGGCAGCAGCTCGGAAAAT	87	300	60
	R:TCCCGGGTGATGTTGTAATCC		300	
IL-8	F:CAAGCAAAAACCCATTCTCCG	99	250	58
	R:CCAGCACAGGAATGAGGCATA		250	
CCL20	F:TTGCTCCTGGCTGCTTTGAT	210	250	60
	R:ATCTGCACACACGGCTAACT		250	
pBD-2	F:TTGCTGCTGCTGACTGTCTG	180	200	62
	R:CTTGGCCTTGCCACTGTAAC		250	
GAPDH	F:GGGCATGAACCATGAGAAGT	230	200	60
	R:AAGCAGGGATGATGTTCTGG		200	
RPL-19	FAACTCCCGTCAGCAGATCC	147	250	60
	R:AGTACCCTTCCGCTTACCG		250	

* TNF-α, tumor necrosis factor α; IL-1β, interleukin-1β; IL-8, interleukin 8; CCL20, Chemokine (C-C motif) ligand 20; pBD-2, porcine β-defensin 2; GAPDH, Glyceraldehyde-3-phosphate dehydrogenase; RPL-19, Ribosomal Protein L19.

## Data Availability

Research data of this manuscript are available upon reasonable request.
